# The use of PRP in treatment of Achilles Tendinopathy: A systematic review of literature. Study design: Systematic review of literature

**DOI:** 10.1016/j.amsu.2020.04.042

**Published:** 2020-06-01

**Authors:** Mr Imad Madhi, Oliver Emmanuel Yausep, Khadija Khamdan, Dionysios Trigkilidas

**Affiliations:** a Trauma and Orthopaedics Surgery, Wythenshawe Hospital, Manchester, UK; b Wythenshawe Hospital, UK; c South Tyneside Hospital, Newcastle, UK; d University of Indonesia, Jakarta, Indonesia

## Abstract

**Background:**

Chronic Achilles Tendinopathy is a common condition that can be challenging to treat. There are many modalities used as treatment ranging from physiotherapy, injections, shockwave therapy to surgical intervention. Platelet Rich Plasma (PRP) has increased in popularity recently as a treatment option for Achilles Tendinopathy. It contains growth factors that might accelerate healing and speed up the recovery of this condition. Many studies have been conducted in the last few years to assess the effectiveness of this treatment method. However, there was controversy as to whether PRP had a beneficial effect on chronic Achille tendinopathy.

**Aim:**

This systematic review of the literature was conducted to ascertain the efficacy of Platelet Rich Plasma (PRP) as a treatment option in chronic Achilles tendinopathy.

**Methods:**

PRISMA reporting item for systematic review has been used to conduct the selection, Electronic databases included PubMed, EMBASE, Cochrane collaborate, Google scholar, the web of science and Cochrane Library were searched for all RCT, prospective and retrospective studies conducted between January 2010 to February 2019. The quality of each study was evaluated using the Oxford CEBM tool to assess the articles for validity, relevance, and applicability of the results. A total number of 288 were found, and only 11 met the inclusion criteria.

**Results:**

5 Randomised control trials, 4 prospective and 2 retrospective cohort study were included in this systematic review. A total number of 406 patients were treated for non-insertional Achilles tendinopathy of which 230 patients had PRP local injection under Ultrasound guide.

**Conclusion:**

Although many of the retrospective studies suggested an advantage of using PRP, the higher level of evidence studies do not support a significant efficacy. This systematic review showed very promising results from the use of Platelet Rich Plasma demonstrated by a significant improvement in the VAST-A score, but we certainly need decent size randomised control trials to show better and more accurate results.

## Highlights

• Platelet rich plasma (PRP) has increased in popularity as a treatment option for Achilles Tendinopathy as it contains growth factors that might accelerate healing and speed up the recovery of this condition.
• Results of this systematic review showed significant improvement in the VISA-A score of the overall studies 28.9 point, comparing the VISA-A score using PRP with the most recent systematic review studied the effectiveness of heavy eccentric calf training (Murphy et al., 2019) [20].
• Most of the RCTs were characterised by their small sample sizes which might resulted in the insignificant results.

## 1 Introduction

Achilles tendinopathy is a chronic musculoskeletal disorder, commonly involves the mid-portion of the tendon [1]. The risk factors are usually associated with overuse in runners, others include trauma, poor exercise, rheumatoid arthritis, and steroid medications. Aetiology might start as breakdown or micro-tears through the tendon due to chronic or repetitive overload with failure of the normal healing process which results in degenerative tendinosis. The tendinopathy usually involves hypo-vascular area 2–6 cm proximal to the calcaneus insertion [2]. Several treatment methods have been implemented in clinical practice, mainly non-operative treatments such as eccentric strengthening exercises, shockwaves, non-steroidal anti-inflammatory medications, and local steroid injections. However, none of these methods deemed to be successful. In recent years, autologous blood and Platelet Rich Plasma (PRP) have been used and demonstrated promising results. Plate Rich Plasma (PRP) is a highly concentrated platelet content comparing to plasma, it contains several cytokines and proteins that act as cell adhesion molecules and growth factors. These growth factors include 3 isomers of platelet-derived growth factors (PDGFaa, PDGFbb, and PDGFab), 2 of the numerous transforming growth factors- (TGF1 and TGF2), vascular endothelial growth factor, and epithelial growth factor [3]. Recently, there has been an increasing interest in using PRP for different bone and soft tissue pathologies to enhance healing, more specifically in joint osteoarthritis, soft tissue injuries, and chronic tendinopathies. Few studies have been conducted to test the effectiveness of PRP treatment in Achilles tendinopathies, however, results were inconsistent. Therefore, a larger systematic review was conducted to include all related good quality peer-reviewed studies. This systematic review aims to analyse the correlation of functional outcome and imaging results following PRP treatment.

## 2 Materials and methods

Preferred reporting item for systematic review and meta-analysis (PRISMA) has been followed to conduct this systematic review [4].

### 2.1 Search strategy

The electronic databases used to search for the related peer-reviewed articles were from PubMed, EMBASE, Cochrane Collaborate, Google Scholar, the web of science and Cochrane library. The databases were searched between January 2010 to February 2019. The keywords used for the search were “Achilles OR Achilles tendinopathy” and “PRP OR platelet-rich plasma OR Platelet concentrate”. The selected articles were limited to trials on humans and published in English.

### 2.2 Inclusion/exclusion criteria

Participants were adult patients with isolated Achilles tendon disorder diagnosed based on either history and physical examination or imaging. Studies with the following designs randomised controlled trials, prospective and retrospective cohort studies were included. Thus, case series and case reports were excluded.

Studies reporting at least one of the following outcomes: Time to recover (or play), recurrences, patient-reported outcomes (PROMs), pain scales, adverse events, or VISA score. Further assessment of articles validity, relevance and applicability were conducted using Oxford CEBM tool. The primary outcome was the VISA-A score and Ultrasound scan assessment of the tendon thickness pre- and post-treatment (Table 3, Table 4).

### 2.3 Data extraction

**Fig. 1:**
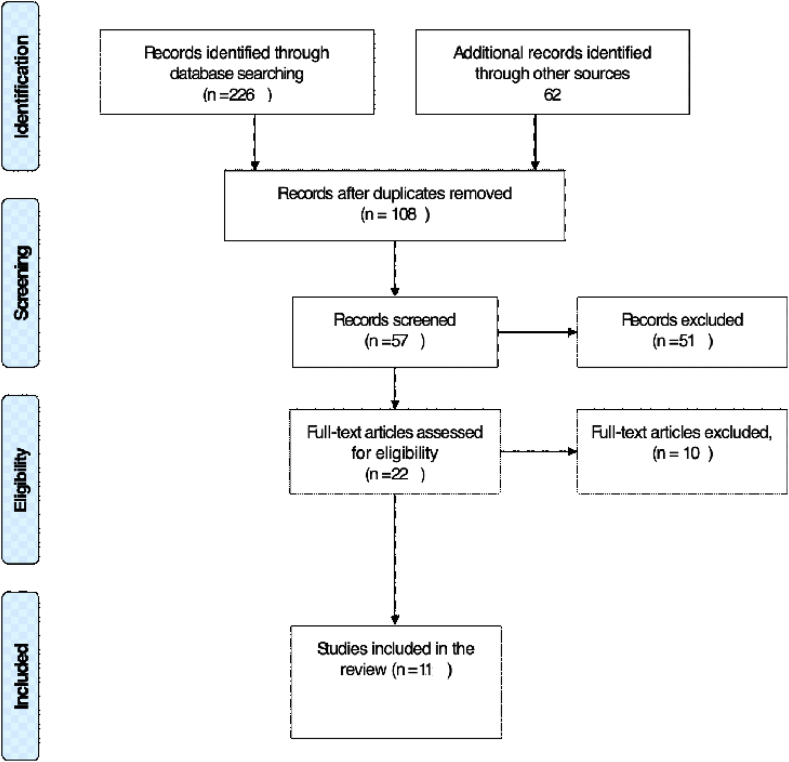
Prisma Flow chart.

The first stage of the search involved the use of the relevant keywords to scan the databases. Initially, the search identified a total number of 288. A total number of 108 articles were identified after removal of duplicated studies. The second stage was to screen the relevant articles by title, the total number of articles related to our subject was 57 articles. Further examination of the full papers found that 22 studies were closely linked to the topic of the systematic review. The third stage was aimed at selecting papers that met the inclusion requirements and the total number of articles chosen was 11. Following selection, a final review of the full articles was conducted by two reviewers and articles did not match the selection criteria were excluded from the study. Fig. 1 PRISMA outflow chart summarises the search strategy for this systematic review.

### 2.4 Quality and appraisal of studies

Oxford CEBM tool was used to assess the articles for validity, relevance and applicability of the results (Table 1). All studies were of high quality and varied slightly in terms of the blinding methods implemented. Some studies, like the one conducted by Kearney et al. (2013) [10] only implemented blinding for data collection, but not for the physicians delivering, or patients receiving treatment. Other studies had created ways of implementing study blinding, such as blindfolding patients in the study by Krogh et al. (2016) [11] and the use of an opaque covering sheath for syringes in the study by de Vos et al. (2010) [12], which revealed statistically significant differences between baseline parameters for primary outcomes but adjusted for them afterwards.

**Table 1:** Oxford CEBM tool.

Articles	Relevance				Validity							Applicability		
	Domain	Determinant	Outcome	Levels of evidence*	Study design	Number of patients	Randomization	Similarity At Baseline	Blinding	Equality Outside Treatment	Accountability	Applicability to Patient	Clinically Important Outcomes	Benefits > Cost?
Albano et al. (2017) [8]	+	+	+	1	RCT	43	+	+	+	+	+	+	+	+
Boesen et al. (2017) [9]	+	+	+	1	RCT	60	+	+	+	+	+	+	+	+
Kearney et al. (2013) [10]	+	+	+	1	RCT	20	+	+	+/-	+	-	+	+	+
Krogh et al. (2016) [11]	+	+	+	1	RCT	24	+	+	+	+	-	+	+	+
de Vos et al. (2010) [12]	+	+	+	2	RCT	54	+	-	+	+	+	+	+	+
(Silvestre et al., 2014) [14]	+	+	+	2	P-Cohort	32	+	-	+	+	+	+	+	+
Gaweda et al. (2010) [15]	+	+	+	2	P-Cohort	15	-	+	-	+	+	+	+	+
Filardo et al. (2014) [16]	+	+	+	2	P-Cohort	27	-	+	-	+	+	+	+	+
Ferrero et al. (2012) [17]	+	+	+	2	P-Cohort	48	-	+	-	+	+	+	+	+
Murawski et al.[18]	+	+	+	4	Case Series	32	-	-	-	+	+	+	+	+
Owens et al. (2011) [19]	+	+	+	4	Case Series	10	-	+	-	+	+	+	+	+

RCT: Randomised controlled trial; + Clearly stated in the article; - Not being done in the article; Not clearly stated; * Levels of evidence based on The Oxford Centre of Evidence Based Medicine 2011.

+/- blinding, and no blinding, were done in intervention, and data collection, respectively.

## 3 Study characteristics

The characteristics of the studies selected for the systematic review were summarised in Table 2. Only 5 randomised control trials, 4 prospective, and 2 retrospective cohort studies were included. A total number of 406 patients were treated for non-insertional Achilles tendinopathy, of which 230 patients had PRP local injection under Ultrasound guide. The mean age of participants was 45.6 years. All RCTs compared the use of PRP to saline injection apart from one RCT compared the difference between PRP and adipose-derived stromal vascular injections. PRP preparation methods were homogenous across the studies selected for this systematic review, however, there were some variations in the amount of the PRP injection and its frequency.

**Table 2:** Summary: Characteristics of the studies included demographics, Treatment Regimen and Follow up.

Study (year)	Study design	Inclusion criteria	Patient number	Age PRP group	Gender	Treatment regimen	Outcome measure	Follow up
(Albano et al., 2017) [8]	RCT	Clinically diagnosed chronic mid-portion Achilles	22/21 43	47.8 ± 5.1		PRP total of 4 ml injected ultrasound-guided	VAS MRI	24
(Boesen et al., 2017) [9]	RCT	Clinically and USS midportion Achilles tendinopathy	19/19/19 57	43.1 ± 8.1	57	PRP total of 4 ml injected ultrasound-guided	VAS VISA-A USS	6-12-24-54
(Kearney, Parsons, and Costa 2013) [10]	RCT	Clinically and USS midportion Achilles tendinopathy	10/10 20	49.9/47.8	7:13	PRP total of 3–5 ml injected ultrasound-guided	VISA-A EQ-5D	6-12-24
(Krogh et al., 2016) [11]	RCT	Clinically and USS midportion Achilles tendinopathy	12/12 24	46.7 ± 9.0/51.8 ± 9.4	13:11	PRP total of 4 ml injected ultrasound-guided	VISA-A USS	12-24-54
(de Vos et al., 2010) [12]	RCT	Clinically diagnosed chronic mid-portion Achilles tendinopathy	27/27 54	49 ± 8.1	13:14	PRP total of 4 ml injected ultrasound-guided	VISA-A sonography	6-12-24
(Silvestre et al., 2014) [14]	prospective study	Clinically and USS midportion Achilles tendinopathy	32	42	27:5	PRP total of 3–3.5 ml injected ultrasound-guided	PAIN USS	4-8-12-24-54
(Gaweda, Tarczynska, and Krzyzanowski 2010) [15]	prospective study	Clinically and USS midportion Achilles tendinopathy	14	40	6:8	PRP total of 3 ml injected ultrasound-guided	VISA-A USS	6-12-24-54
(Filardo et al., 2014) [16]	prospective study	Clinically and USS midportion Achilles tendinopathy	27	44.6 ± 10.6	22:5	PRP total of 5 ml injected ultrasound-guided	VISA-A EQ-VAS	8–24
(Ferrero et al., 2012) [17]	prospective study	Clinically and USS midportion Achilles tendinopathy	24	38.6		PRP total of 6 ml injected ultrasound-guided	VISA-A USS	3–24
(Murawski et al., 2014) [18]	Retrospective study	Clinically and USS midportion Achilles tendinopathy	33	41	21:11	PRP total of 3 ml injected ultrasound-guided	Sf-12 FAOS MRI	24
(Owens et al., 2011) [19]	Retrospective study	Clinically and MRI midportion Achilles tendinopathy	10	52.1	2:8	PRP total of 6 ml injected ultrasound-guided	FAAM SF-8 MRI	
Total			338/230	45.6				

VISA-A: Victorian assessment of sport ability, USS ultrasound scan, PRP: platelet rich plasma, FAAM: Foot and Ankle Ability Measure, FAOS: Foot and Ankle Outcome Score.

**Table 3:** VISA-A score baseline and following injection.

Study (year)	Patient number	VISA-A Pre-Tx	VISA-A Post-Tx	p
(Boesen et al., 2017) [9]	19/19/19	37.1 ± 6.2	58.1 ± 12.4	<0.05
(Kearney et al., 2013) [10]	10/10	41	76	0.171
(Krogh et al., 2016) [11]	12/12	31.7 ± 20.7	35.1 ± 20.7	0.341
(de Vos et al., 2010) [12]	27/27	46.7	68.4	0.868
(Gaweda et al., 2010) [15]	14	24	92	<0.05
(Filardo et al., 2014) [16]	27	49.9 ± 18.1	84.3 ± 17.1	>0.05
(Ferrero et al., 2012) [17]	24	58 ± 16	77 ± 12	0.001
Mean:	Total no 133	41.2	70.12

**Table 4:** Ultrasound and MRI changes baseline and following the injection.

Study (year)	Patient NO	PRE-USS	POST-USS	PRE-MRI	POST-MRI
(Albano et al., 2017) [8]	22/21	8.4 ± 2.2	8.9 ± 3.6 P 0.395	8.8 ± 3.5	9.3 ± 3.4 P 0.290
(Boesen et al., 2017) [9]	19/19/19	8.3 ± 1.4	5.8 ± 1.1 P < 0.05		
(Krogh et al., 2016) [11]	12/12	9.9 ± 2.4	10.7 ± 2.4 P = 0.030		
(de Vos et al., 2010) [12]	27/27	9.8	9		
(Ferrero et al., 2012) [17]	24	15 ± 5	10 ± 3 P = 0.004		

Following PRP preparation, the PRP was injected under ultrasound guidance except in one study that preferred Magnetic Resonance Imaging to identify the area of injection. Most studies injected PRP in three different places of the tendon to ensure good coverage of the affected area and have a follow-up duration of up to one year. To obtain a comparable outcome, study results at a follow-up duration of 24 weeks were considered in this systematic review. Primary outcome results for most of the studies were the VISA-A score as well as Ultrasound scan assessment of the tendon thickness pre- and post-treatment (Table 3, Table 4).

## 4 Results

To evaluate the substantial change in the VISA-A ranking, Wilcoxon signed-ranked pre-and post-treatment tests were used. It is a non-parametric statistical method used to compare two similar samples or repeated measurements. Also, a simple descriptive analysis using the RevMan 5 programme was used to evaluate the total means and P-values.

Patient means baseline VISA-A score was 41.2 which improved to 70.12 after treatment, the mean difference between VISA-A score was 28.9 points which were substantial compared to other non-operative care approaches. Overall P-value of 0.018, which is considered statistically important.

Ultrasound analysis results have not been statistically evaluated as it is subjective to measurement variance between assessors. However, if we compare the results of the ultrasound scan after treatment in (Table 3), we can see the difference in the results reported as 3 studies recorded a decrease in overall thickness and vascularity while the other 2 studies (Albano et al., 2017 [8]; Krogh et al., 2016 [11]) reported an increase in tendon thickness.

## 5 Discussion

This systematic review aims to compare the effectiveness of PRP treatment of Achilles tendinopathy to other modalities of treatments. Achilles tendinopathy occurs most commonly after foot and ankle overuse, which results in pain, swelling and decrease activity.

The treatment has been challenging and various non-operational methods have been commonly offered in clinical practice, but to date, none has been considered effective. Also, surgical treatments including debridement or tendon transfer have not shown any promising results. PRP therapy has been one of the fast-developing topics over the last few years and its effect on soft tissue repair. Recent studies demonstrated its effectiveness in tendon repair. The role of PRP in tendon repair is by activation of the coagulation cascade and the release of multiple factors. Subsequently, injured tendon's tenocytes, leucocytes and local stem cells will be recruited by the factors released by the coagulation cascade promoting the healing pathway. Also, PRP is a collagen couple that plays a key role in restoring normal tissue structure and function [5]. General techniques used in PRP preparation begin with the addition of citrate to the entire blood to bind ionised calcium and inhibit the cascade of coagulation. Followed by centrifugal steps to separate red and white blood cells from plasma and platelets. A second centrifugal may be used to concentrate the platelets and separate the PRP from the low-platelet plasma. To allow the treatment to be given to the affected site, the PRP must be clotted. Multiple commercial systems are now available, some of which use bovine thrombin to activate the cascade of clotting [6]. Outcome measures to assess the effectiveness was by comparing the VISA-A score and radiological changes pre- and post-treatment. Results grouped according to the intervention and the outcome measures, as 8 studies have used VISA-A score as a functional outcome measure, and 5 studies used ultrasound results in their outcome measure. VISA-A score numeric rating scale score range from 0 to 100 points aims to evaluate the severity of symptoms for patients with chronic Achilles tendinopathy and has been considered as a reliable tool to assess outcome following treatment. The VISA-A has had the MCID reported for insertional Achilles tendinopathy with an improvement of 6.5 points reflecting a meaningful improvement for the patient [7]. Results of this systematic review showed significant improvement in the VISA-A score of the overall studies by 28.9 points, which could have been higher if we excluded the results from the study done by (Krogh et al., 2016 [11]) as his trial on 24 patients showed no significant difference after short 3 months follow-up.

The VISA-A score following PRP usage was substantially higher compared to research using intense eccentric calf training (Murphy et al., 2019 [20]) and thus PRP can be a more successful treatment. Comparing outcome results from the radiological evaluation showed some changes in the thickness of tendon with decrease vascularity as reported by (Boesen et al., 2017 [9]; de Vos et al., 2010 [12]; De Jonge et al., [13] and Ferrero et al., 2012 [17]).

Despite this, work by Albano et al., 2017 [8] and Krogh et al., 2016 [11] which showed a slight increase in tendon thickness after 3 months follow up (Table 3). Therefore, the VISA-A score and ultrasound result showed a marked increase in pre-and post-treatment in more than 50% of the studies selected for this systematic review. Nevertheless, it is an early stage of exploring the effectiveness of this new treatment method, and the currently available clinical evidence regarding PRP treatment insertional Achilles tendinopathy was collected either from low-quality clinical evidence or very small trial. The majority of the randomised control trials compared PRP to either saline injection or dry injection, which might contribute to the good outcome in these patients. Saline and dry injections stimulate bleeding and inflammation of the affected part of the Achilles tendon which might stimulate healing, this is likely to affect the overall outcome results of these RCTs.

## 6 Conclusion

Studies collected for this systematic review have borne certain limitations, as some studies have been cohort-controlled and non-randomised. Also, most randomised control experiments had a small sample size. Although many of the retrospective studies suggested an advantage of using PRP, the higher level of evidence studies do not support a significant efficacy. Interestingly, this systematic review showed very promising results from the use of Platelet Rich Plasma demonstrated by a significant improvement in the overall VAST-A score. Future research will benefit from a reasonable size randomised control trial to produce more consistent outcomes.

## Ethical approval

This study did not involve any patients.

## Funding

None.

## Author contribution

Imad Madhi, design data collection, data analysis or interpretation and writing the paper. Oliver Emmanuel Yausep data analysis or interpretation. Khadija Khamdan writing the paper. Dionysios Trigkilidas design, data collection, data analysis or interpretation and writing the paper.

## Registration of research studies

1. Name of the registry: Research Registry.

2. Unique Identifying number or registration ID: reviewregistry809.

3. Hyperlink to the registration (must be publicly accessible): https://www.researchregistry.com/browse-the-registry#registryofsystematicreviewsmeta-analyses/

## Guarantor

Imad Madhi MBChB, MCh (Tr&Orth), MRCS.

Dionysios Trigkilidas MBChB, FRCS (tr & Orth).

## Provenance and peer review

Not commissioned, externally peer reviewed.

## Declaration of competing interest

None.
